# Acute and delayed neuroinflammatory response following experimental penetrating ballistic brain injury in the rat

**DOI:** 10.1186/1742-2094-4-17

**Published:** 2007-07-02

**Authors:** Anthony J Williams, Hans H Wei, Jitendra R Dave, Frank C Tortella

**Affiliations:** 1 Walter Reed Army Institute of Research, Dept. of Applied Neurobiology, Silver Spring, MD, USA

## Abstract

**Background:**

Neuroinflammation following acute brain trauma is considered to play a prominent role in both the pathological and reconstructive response of the brain to injury. Here we characterize and contrast both an acute and delayed phase of inflammation following experimental penetrating ballistic brain injury (PBBI) in rats out to 7 days post-injury.

**Methods:**

Quantitative real time PCR (QRT-PCR) was used to evaluate changes in inflammatory gene expression from the brain tissue of rats exposed to a unilateral frontal PBBI. Brain histopathology was assessed using hematoxylin and eosin (H&E), silver staining, and immunoreactivity for astrocytes (GFAP), microglia (OX-18) and the inflammatory proteins IL-1β and ICAM-1.

**Results:**

Time course analysis of gene expression levels using QRT-PCR indicated a peak increase during the acute phase of the injury between 3–6 h for the cytokines TNF-α (8–11 fold), IL-1β (11–13 fold), and IL-6 (40–74 fold) as well as the cellular adhesion molecules VCAM (2–3 fold), ICAM-1 (7–15 fold), and E-selectin (11–13 fold). Consistent with the upregulation of pro-inflammatory genes, peripheral blood cell infiltration was a prominent post-injury event with peak levels of infiltrating neutrophils (24 h) and macrophages (72 h) observed throughout the core lesion. In regions of the forebrain immediately surrounding the lesion, strong immunoreactivity for activated astrocytes (GFAP) was observed as early as 6 h post-injury followed by prominent microglial reactivity (OX-18) at 72 h and resolution of both cell types in cortical brain regions by day 7. Delayed thalamic inflammation (remote from the primary lesion) was also observed as indicated by both microglial and astrocyte reactivity (72 h to 7 days) concomitant with the presence of fiber degeneration (silver staining).

**Conclusion:**

In summary, PBBI induces both an acute and delayed neuroinflammatory response occurring in distinct brain regions, which may provide useful diagnostic information for the treatment of this type of brain injury.

## Background

The brain has been traditionally described as an "immune-privileged" organ due to its isolation from the peripheral immune system by the blood-brain barrier (BBB). However, recent brain injury studies have indicated that resident brain cells are capable of synthesizing a wide variety of pro-inflammatory mediators necessary for mounting a neuroinflammatory response [1]. Injury to the brain induces expression and release of small BBB permeable cytokines and chemokines that can stimulate the peripheral immune system and attract peripheral inflammatory leukocytes to the site of injury [2]. Cytokine-mediated intracellular signalling also induces expression of intercellular adhesion molecules and stimulation of cerebral leukocyte infiltration across the BBB [3,4]. Leukocyte accumulation within the injured brain has been purported to contribute to the injury process due to release of cytotoxic substances including reactive oxygen species and other pro-inflammatory mediators [2]. Although phagocytic clearing of cellular debri is inherent to the natural healing process, release of pro-inflammatory molecules can potentially induce further stress to the already compromised penumbral regions of the injured brain (i.e. bystander injury) in a feed-forward cascade of inflammatory cell-mediated injury [2,5].

Aside from stimulation of the peripheral immune system, brain injury induces activation of resident glial cells that participate in the inflammatory response. In particular, microglial cells are strongly activated following injury and play an important role in phagocytosis of injured brain tissue [6]. Other cells such as astrocytes are also activated exhibiting a hallmark increase in glial fibrillary acidic protein (GFAP) expression [7]. Astrocytes are known to be a source of pro-inflammatory cytokines [8] and in the advanced stages of injury progression form a glial scar inhibitory to neural regeneration [9]. However, reactive astrocytes generally serve a protective role during the early stages of injury and are responsible for maintaining physiological homeostasis and free radical elimination during periods of energy deprivation [7,10].

The current research focuses on the neuroinflammatory response out to 7 days following a penetrating ballistic-like brain injury in the rat [11-13] out to 7 days following the initial insult. Results indicate 1) a rapid upregulation of pro-inflammatory cytokine and cell adhesion molecule genes within the first few hours post-injury, 2) both an acute and delayed inflammatory cell response occurring in distinct brain regions 3) a differential temporal profile of astrocyte as compared to microglial activation, 4) a distinct glial response in the thalamus predictive of delayed fiber degeneration within this same region. We conclude that PBBI involves a robust neuroinflammatory response that may help guide the potential management of this type of injury.

## Methods

Male Sprague-Dawley rats (250–300 g; Charles River Labs, Raleigh, VA) were used in all studies. All procedures were approved by the Walter Reed Army Institute of Research Animal Care and Use Committee. Research was conducted in compliance with the Animal Welfare Act and other federal statutes and regulations relating to animals and experiments involving animals and adhered to principles stated in the Guide for the Care and Use of Laboratory Animals, NRC Publication, 1996 edition. Animals were housed individually under a 12 h light/dark cycle (lights on at 0600) in a facility accredited by the Association for Assessment and Accreditation of Laboratory Animal Care International.

### Penetrating ballistic brain injury model

The Dragonfly Model # HPD-1700 Variable Pressure Waveform Generator and rat PBBI probe (Dragonfly Inc., WV) were used to simulate a right frontal ballistic injury of a high-velocity 7.62 mm North Atlantic Treaty Organisation (NATO) round to the rat brain [11]. In brief, rats were anesthetized (2% isoflurane delivered in oxygen) and positioned in a stereotaxic frame for probe insertion (Kopf, Tujunga, CA). Body temperature was maintained normothermic (37 ± 1°C) throughout the surgical procedure by means of a homeothermic heating system (Harvard Apparatus, South Natick, MA). The scalp was incised along the mid-line and a 1 mm diameter burr hole was created to expose the right frontal pole of the brain (+4.5 mm AP, +2 mm ML to bregma). The PBBI probe was mounted to a stereotaxic arm and fixed at an angle of 50 degrees from vertical and 25 degrees counter-clockwise from the anterior-posterior axis. The probe was then advanced with a micromanipulator (~2 mm/s) along this axis through the cranial window into the right frontal cortex, through the dorsal aspect of the caudate putamen, and into the amygdalar region of the brain. The tip of the PBBI probe travelled a total distance of 1.2 cm from the surface of the brain.

A 10% severity level of injury was used in the current study, which represents a moderate-severe but survivable level of injury associated with consistent and reproducible histopathological damage [11,13]. PBBI was induced by delivery of a pressure pulse to rapidly inflate and deflate the PBBI balloon (to a diameter of 6.33 mm, equal to 10% of total rat brain volume). Immediately following inflation, the probe was manually retracted from the brain, the cranial opening sealed with sterile bone wax, and the incision closed with 4-0 nylon suture followed by administration of a topical antibiotic. Following surgery, animals were placed in a warm chamber maintained by a circulating water-bath heating system (Gaymar Indust., Orchard Park, NY) until recovery from anesthesia. Food and water were provided *ad libitum *post-operatively. Sham animals were not subjected to probe insertion but otherwise received all surgical manipulations.

### Histopathology

At the indicated post-injury endpoint, animals were anesthetized with ketamine/xylazine (70 and 6 mg/kg, i.p., respectively) and transcardially perfused with phosphate buffered saline (PBS; pH 7.4 at room temperature) followed by ice-cold 4% paraformaldehyde. Brains were extracted, immersed in 4% paraformaldehyde for 6 h and then transferred to 0.1 M phosphate buffer containing 20% sucrose (pH 7.4, 4°C). All brain tissue was sent to FD Neurotechnologies (Baltimore, MD) for histopathological staining.

Coronal brain sections (40 μm thick) were cut through the cerebrum from +4.0 to -7.0 mm AP to bregma with serial sections collected at 1 mm intervals. Silver staining was performed using the FD Neurosilver™ Kit I (FD NeuroTechnologies, Baltimore, MD) according to the manufacturer's instructions to detect fiber degeneration. Hematoxylin & eosin (H&E) was used for morphological assessment of injury and detection of inflammatory cells including polymorphonucleocytes (neutrophils), monocytes, and macrophage-like cells (defined by large irregular cytoplasm) [14]. Antibodies against OX-18 were used for detection of activated microglia and GFAP for detection of activated astrocytes [11]. In brief, following inactivation of endogenous peroxidase activity, biotin, biotin-binding proteins, and lectins, sections were incubated free-floating in PBS containing 1% normal horse serum (Vector Labs, Burlingame, CA), 0.3% Triton X-100 (Sigma, St. Louis, MO) and polyclonal rabbit anti-cow GFAP IgG (1:20,00; Z0334, DAKO, Glostrup, Denmark) or monoclonal mouse anti-OX18 IgG (1:6,000; MCA51R, Serotec, Raleigh, NC) for 3 days at 4°C. The immunoreaction product was then visualized according to the avidin-biotin complex method of Hsu et al. (1981) [15] with Vectastin Elite ABC kit (Vector Laboratory, Burlingame, CA). After dehydration in ethanol and clearing in xylenes, all sections were coverlipped in Permount^® ^(Fisher Scientific, Fair Lawn, NJ). All tissue samples were evaluated by light microscopy.

### Tissue processing for gene expression studies

**Figure 1:**
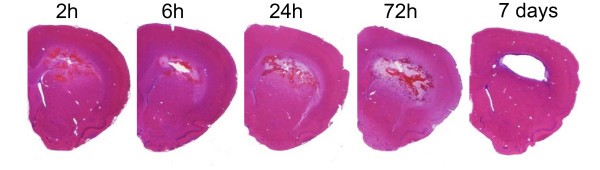
**PBBI histopathology**. Time course change in brain pathology following PBBI (H&E) leading to eventual formation of a large intracranial cavity by day 7 post-injury. Representative coronal sections (1 mm anterior to bregma) are shown from the ipsilateral hemisphere following PBBI in rats.

At each given endpoint, rats were deeply anesthetized with ketamine/xylazine (70 and 6 mg/kg, i.p., respectively) and brains harvested for analysis. A 2 mm section was dissected from the core lesion of each rat brain (1–3 mm anterior to bregma, as shown in Figure 1), divided into hemispheric sections contralateral and ipsilateral to the primary injury, rapidly frozen on dry ice, and stored at -70°C until RNA extraction. Frozen brain tissue was homogenized in QIAzol ™ lysis reagent and total RNA extracted using Qiagen RNeasy Liquid Tissue Mini Kit according to the manufacturer's instructions (Qiagen Science, Germantown, MD). RNA purity and concentration were determined spectrophotometrically by calculating the ratio between the absorbance at 260 nm and 280 nm. The absorbance ratio for all samples ranged between 1.8 and 2.1. The quality of RNA for all samples was confirmed by resolving on a 1.5% formaldehyde agarose gel.

### Real time (RT)-PCR

Quantitative RT-PCR was performed with ABI Prism 7000 sequence detection system (PE Applied Biosystems) as previously described [16] using TaqMan™ Universal PCR Master Mix (*AB *Applied Biosystems) as per manufacturer's specifications. Amplification conditions included 2 minutes at 50°C and 10 minutes at 95°C, and then run for 40 cycles at 95°C for 15 seconds and 60°C for 1 minute. PCR primers and TaqMan™ probes were designed using Primer Express 2.0 Software and synthesis was performed by *AB *Applied Biosystems. Primer/probe sequences used are as previously reported [16]. Data are presented as relative induction of each cytokine or cellular adhesion molecule normalized to ribosomal protein L32 (RPL32).

### Statistical analysis

Quantitative data (i.e. RT-PCR gene expression levels) were analyzed using paired t-tests between the ipsilateral and contralateral brain hemispheres. The contralateral hemisphere served as an internal control for each brain sample. Data are presented as mean ± standard deviation. P values < 0.05 were considered significant.

.

## Results

### General pathology

PBBI induced a unilateral lesion to the right brain hemisphere predominately involving the frontal cortex and striatum. A hemorrhagic core lesion developed along the injury track reaching maximal size by 24–72 h with formation of an intracranial cavity by day 7 post-injury (Fig. 1).

### Inflammatory gene expression profile

**Figure 2:**
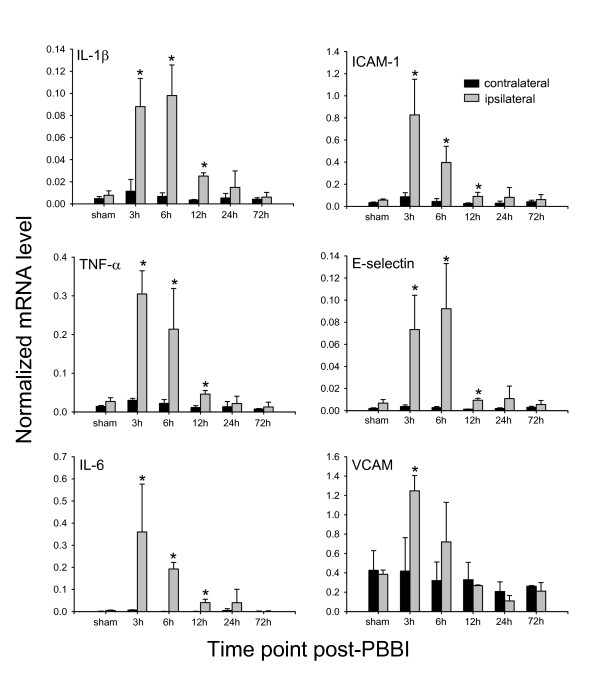
**Inflammatory gene expression**. Change in mRNA expression levels of 6 pro-inflammatory genes in injured brain tissue from 3 to 72 h following PBBI (QRT-PCR). Data presented as mean ± standard deviation (n = 3/group). * P < 0.05 as compared to contralateral hemisphere.

QRT-PCR was used to verify and track the time course changes of 3 cytokine (TNF-α, IL-1β, and IL-6) as well as 3 cellular adhesion molecule (ICAM-1, VCAM, and E-selectin) mRNA levels following PBBI (Fig. 2). These specific inflammatory genes were chosen for direct comparison to previously published time course data in a rat focal brain ischemia model using the same QRT-PCR primer and probes [16].

**Figure 3:**
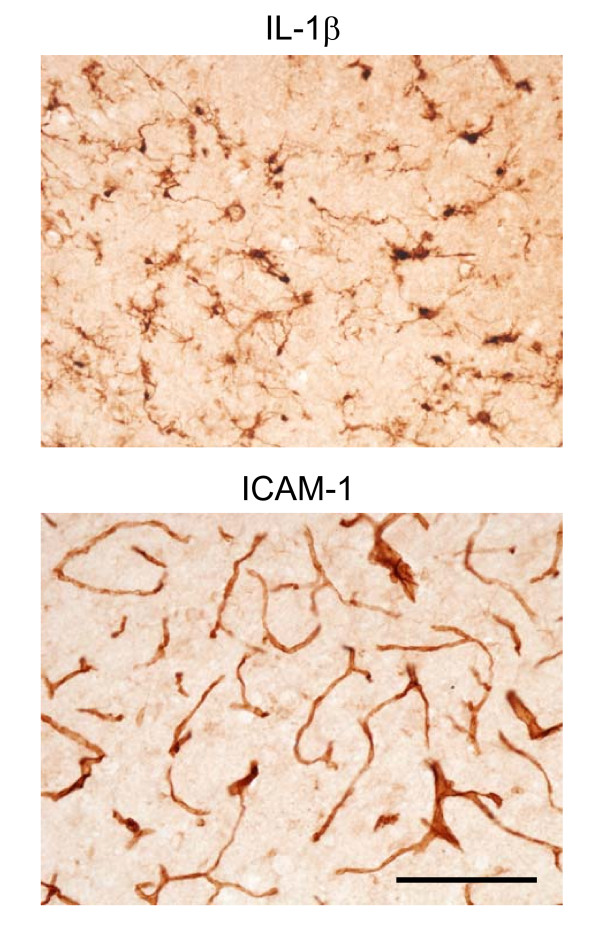
**IL-1β and ICAM-1 immunoreactivity**. IL-1β was most notable in ramified cells surrounding the lesion (upper panel). ICAM-1 was only observed in the vasculature surrounding the immediate lesion track (lower panel). Bar = 100 μm

All 6 inflammatory genes were significantly up-regulated between 3–12 h post-injury with peak expression levels occurring between 3–6 h. The increase in expression subsided by 24–72 h post-injury. Peak increases in gene expression as compared to sham tissue were as follows: TNF-α (8–11 fold), IL-1β (11–13 fold), IL-6 (40–74 fold), VCAM (2–3 fold), ICAM-1 (7–15 fold), and E-selectin (11–13 fold). In comparison to focal ischemic brain injury, inflammatory gene expression exhibited elevated and more rapid increases in mRNA expression levels, as indicated by IL-1β and ICAM-1 (Fig. 3).

Changes in IL-1β and ICAM-1 expression were also verified by immunostaining brain tissues with antibodies for the respective proteins. IL-1β immunoreactive cells demonstrated dark nuclear and cytoplasmic staining including ramified processes typical in morphology to glial cells (Fig. 3). In contrast, ICAM-1 expression was restricted to vascular cells also surrounding lesion sites (Fig. 3).

### Inflammatory cell response (H&E)

**Figure 4:**
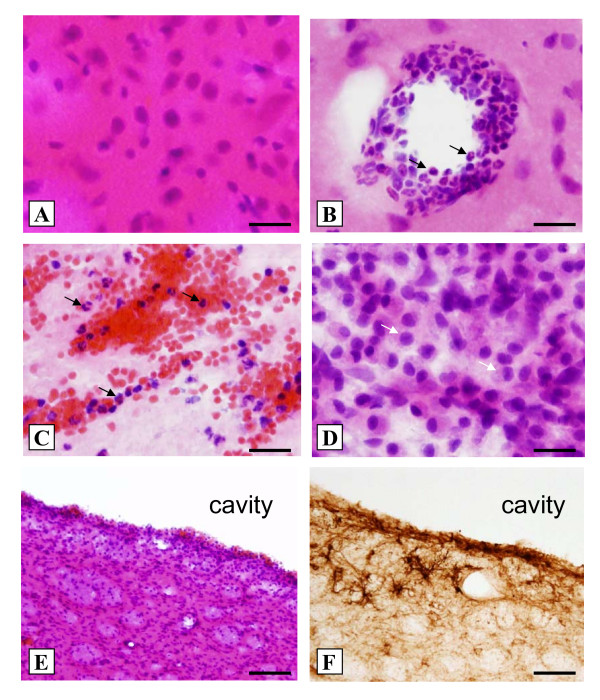
**Inflammatory leukocyte infiltration**. Neutrophils (black arrows) were present as early as 6 h post-injury lining vessel walls (B, H&E) with intraparenchymal invasion by 24 h and beyond (C, H&E) as compared to sham tissue (A, H&E). A dense array of large macrophage-like cells (white arrows) were prominent by 72 h, particularly along the immediate border of the PBBI lesion (D, H&E). By day 7 an intracranial cavity had formed surrounded by a dense cellular infiltrate (E, H&E) and large, highly ramified astrocytes (F, GFAP). Bar = 20 μm (A-D), 100 μm (E-F).

The normal (sham) rat brain did not exhibit any distinctive evidence of inflammatory cell infiltrate (Fig. 4A). In the PBBI animals, intravascular neutrophil clustering appeared as early as 6 h post-injury (Fig. 4B) surrounding the primary lesion site with migration of neutrophils into the core lesion by 24–72 h (Figure 4C). By 72 h, parenchymal monocytes/macrophages were the dominant cell observed throughout the core lesion (Figure 4D). By day 7 most of the inflammatory infiltrate was confined to the immediate region of the primary lesion site (Figure 4E) along with a band of reactive gliosis (Figure 4F).

### Gliosis (astrocytes and microglia)

**Figure 5:**
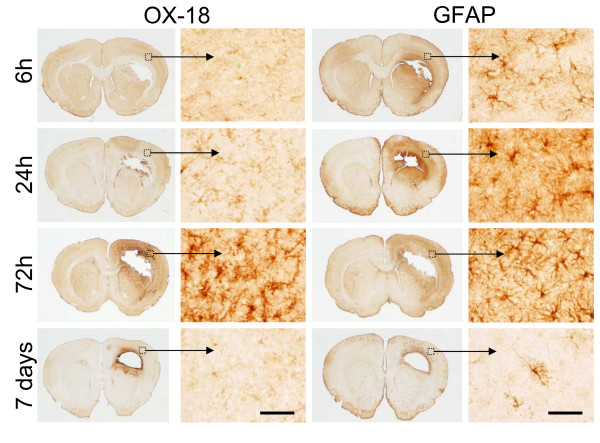
**Inflammatory gliosis**. Representative coronal brain sections (1 mm anterior to bregma) indicating the time course change in activated microglial cells (OX-18 immunoreactivity) and reactive astrogliosis (GFAP immunoreactivity) surrounding the primary lesion site. Bar = 100 μm.

Activated microglia appeared as highly ramified cells reactive for OX-18 (Fig. 5). In the peri-lesional regions of the cortex and striatum, microglia exhibited immunoreactive processes starting 24 h post-injury with maximal activation and "bushy" morphology by 72 h with almost complete resolution by day 7 (Fig. 5) except for a strong band of reactivity immediately surrounding the lesion at day 7. The peri-lesional region also exhibited the presence of highly ramified GFAP-immunoreactive astrocytes with a more rapid post-injury expression profile as compared to microglial activation (Fig. 5 & Table 1). Strongly reactive astrocytes with hypertrophic soma and thick processes were observed as early as 6 h post-injury with a progressive increase in cell density out to 72 h (Fig. 5). By day 7, the peri-lesional regions were sparsely reactive for astrocytes with a similar band of reactive cells immediately surrounding the lesion (Figs. 4F &5) as observed for microglia. The GFAP and OX-18 images shown in Fig 5 are from the same animal at each respective time point (4–6 animals were evaluated at each post-injury time point).

**Table 1:** Glial cell reactivity

**Time Point**	**GFAP**	**OX-18**
**Sham**	0	0
**6 h**	+ +	+
**24 h**	+ +	+
**72 h**	+ + +	+ + +
**7 days**	+	+

Degree of immunoreactivity for astrocytes (GFAP) and microglial cells (OX-18) in brain tissue surrounding primary PBBI lesion (0 = none, + minimal, + + moderate, and + + + strong reactivity). Based on evaluation of 4–6 animals per post-injury time-point.

### Delayed thalamic inflammation

**Figure 6:**
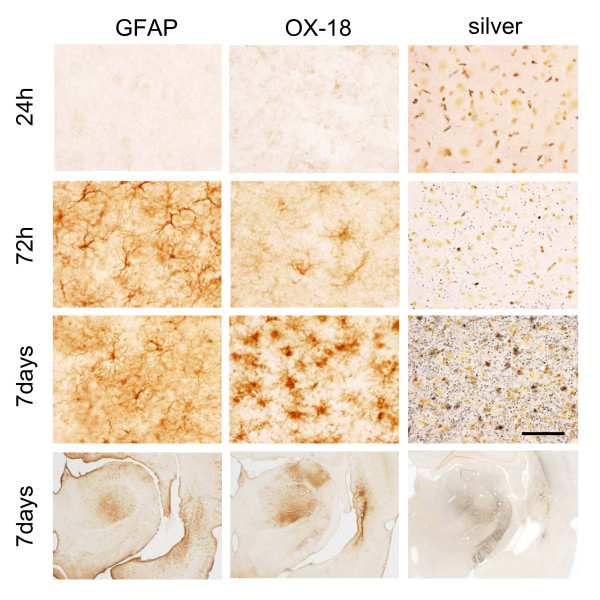
**Delayed inflammation**. Delayed inflammatory response of the thalamus to PBBI. Both activated astrocytes (GFAP) and microglial cells (OX-18) were observed from 72 h to 7 days post-injury and corresponded to an increase in fiber degeneration (silver staining). Bar = 100 μm.

There was no histologic evidence of injury in the ipsilateral hemisphere remote from the core lesion or in the contralateral hemisphere through 24 h following PBBI. However, fiber degeneration (silver staining) became apparent by 72 h in remote ipsilateral brain regions such as the thalamus (Fig. 6), internal capsule, and cerebral peduncle and was associated with strong glial reactivity (Fig. 6). In particular, at the early stages of fiber degeneration in the thalamus, GFAP reactive cells were notable (72 h) and remained through day 7 (Figure 6). In contrast, the presence of activated microglia was minimal at 72 h and became prominent by day 7, particularly in regions of intense silver reactivity (Fig. 6).

## Discussion

The current data indicate that PBBI induces a biphasic pattern of neuroinflammation in anatomically distinct regions of the brain, progressing from the site of the "primary lesion" to a "delayed injury" occurring in remote brain structures such as the thalamus. The acute neuroinflammatory response was associated with upregulation of pro-inflammatory cytokines and cellular adhesion molecules with peak increases in mRNA levels between 3–6 h post-injury followed by inflammatory cell activation and recruitment into the primary lesion. The early post-injury upregulation of cytokines such as IL-1β appeared to occur predominately in glial-type cells, corresponding to the early activation of astrocytes. Vascular expression of cellular adhesion molecules (e.g. ICAM-1) likely contributed to the intravascular collection of peripheral neutrophils by 6 h [17], a possible pathological response leading to further injury due to microvascular occlusion [3]. Inflammatory cell infiltrate was notable in the ensuing post-injury days and by 72 h a dense phagocytic infiltrate was present within the primary lesion surrounded by a maximal expression of activated cortical/striatal microglia. By day 7 the primary lesion was largely resolved, replaced by an intracranial cavity and surrounded by a rim of strong glial reactivity. In contrast, a second phase of neuroinflammation occurred in the thalamus as indicated by glial cell activation, concomitant with an extensive degree of fiber degeneration in this region.

The rapid increase in inflammatory gene expression following PBBI may have significant implications on the therapeutic management of this type of neuroinflammatory response. In comparison, previous studies from our own lab have indicated a delayed increase in pro-inflammatory gene expression induced by 2 h of transient middle cerebral artery occlusion (MCAo) in rats with peak increases between 6–24 h for the same pro-inflammatory genes evaluated in the current study [16]. The rapid activation of cytokine gene upregulation appears to be more pronounced in traumatic versus ischemic type injuries as demonstrated with PBBI and supported by data from other rat TBI studies [18-21]. In particular, both IL-1β and TNF-α mRNA levels have been shown to peak 1–3 h following experimental TBI in rats (Fan, 1996)[20,21]. The clinical implication for these differences in inflammatory gene expression between traumatic versus ischemic injury may be dramatic. For example, attenuation of the nuclear factor κB (NF-κB) mediated pro-inflammatory gene response through proteasome inhibition offers a significant neuroprotective recovery from transient MCAo in rats [22,23]. Importantly, the 6–10 h therapeutic treatment window associated with proteasome inhibition in the MCAo model directly correlated to peak levels of inflammatory gene expression [24-26]. Given the rapid increase in inflammatory gene expression following PBBI, we would hypothesize that neuroprotection therapies targeting the neuroinflammatory gene response (e.g. regulators of NF-κB activity), would offer a shorter therapeutic window as compared to focal ischemic injury, thereby requiring quicker therapeutic intervention.

Another interesting aspect of this study was the differential response of astrocytes and microglia to PBBI. In the cortical regions surrounding the primary lesion astrocytes were rapidly activated and remained strongly reactive through 72 h post-injury. In contrast, microglia were slower to react and did not become fully reactive until 72 h post-injury. A similar but delayed pattern of glial cell activation was observed in the thalamus as well with maximal reactive astrogliosis occurring at 72 h post-injury followed by maximal microglial activation at day 7. These data would suggest fundamentally different roles for these two types of cells in response to brain injury possibly reactive to distinct triggering mechanisms (as discussed below). Although the presence of glial activation in the injured brain certainly represents a disturbance of normal brain physiology or predictor of a pathological condition, it is still controversial as to whether reactive gliosis is harmful or beneficial to the acutely injured brain [27,28].

The rapid activation of astrocytes surrounding the primary PBBI lesion likely reflects a response to the upregulation of cytokine signalling [27] but may also be aggravated by changes in the extracellular matrix such as increases in K^+ ^due to peri-infarct depolarizations, a common phenomenon following PBBI [11]. Astrocytes react to changes in the extracellular environment through uptake of extracellular glutamate, K+ buffering, and elimination of free radicals which may be crucial for neuronal survival during the acute post-injury period [7,10]. Astrocytes, in general, are more resistant than neurons during periods of energy failure or following toxic insult. However, overextension of the protective capability of the astroglial response will ultimately lead to cell death. In fact, injury-induced astrocytic swelling is responsible for production and release of excitotoxic agents including quinolinic acid and glutamate and ultimately a loss of normal homeostatic function that may exacerbate neuronal damage [28,29]. Direct astrocytic malfunction or loss of viability has been coupled to the subsequent death of neurons within the surrounding environment [30].

In contrast to astrocyte activation, widespread microglial reactivity in cortical regions surrounding the primary lesion was not observed until 72 h following PBBI. The triggering mechanism for this delayed response is unknown, although it does correspond to the mass migration of large amaeboid macrophages into the primary lesion site. Microglial cells are the resident macrophages of the CNS of myeloid origin that can exhibit a wide spectrum of stimulus-specific responses [31]. Specifically, microglial cells become activated through cell-surface stimulation of toll-like receptors [31], ultimately leading to the phagocytic activity of these cells, but are also modulated through activation of purinergic [32,33] and CD40 receptors [34]. Similar to astrocytes, microglia may be beneficial or harmful to the injured brain depending on their specific environmental context including degree and length of activation or post-injury time point of reactivity. Microglia can be beneficial to the injured brain through production of trophic factors and phagocytic resolution of the lesion [30] although production of pro-inflammatory cytokines and reactive oxygen species may be harmful to surrounding tissues [31]. The definitive role of microglia in the injured brain remains to be elucidated although experimental therapies that reduce microglial reactivity following PBBI have been associated with improved pathological and functional outcome [35].

Two distinct and divergent temporal neuroinflammatory profiles were also captured with the PBBI model. The first being the rapid neuroinflammatory response within the core lesion characterized by a strong gliotic response coupled with mass infiltration of peripheral inflammatory cells. In contrast, the neuroinflammatory response in the thalamus involved primarily a gliotic response void of the marked infiltration of peripheral inflammatory cells as seen in the primary lesion. Delayed thalamic degeneration, similar to that observed following PBBI, has also been observed in other focal brain injury models including injuries induced by cortical ablation, infarction, or trauma [11,36-40]. Similarly, loss of thalamic volume has been observed in human stroke and TBI patients exhibiting cortical lesions [41,42]. This pattern of delayed thalamic injury and associated neuroinflammatory response is of particular interest as it represents a delayed target of neuroprotective intervention aimed at preserving thalamic integrity and improving patient outcome [39]. In particular, GFAP may hold promise as a potential therapeutic biomarker of delayed brain injury as it is expressed prior to thalamic injury, as indicated following PBBI, and is specific for glial cells of the CNS [43]. Increases in GFAP concentrations have been observed in CSF samples following human stroke [44] and in blood samples of severe TBI patients [45] although expression has not be correlated to delayed injury processes such as thalamic degeneration.

## Conclusion

In conclusion, this is the first report to indicate the unique neuroinflammatory time course profile of a penetrating ballistic-like injury to the brain. The acute neuroinflammatory response occurred very rapidly post-injury with a peak upregulation of inflammatory genes occurring on the order of several hours before that reported for similar responses in focal ischemic injury models. Glial reactivity surrounding the primary lesion, as indicated from the presence of highly ramified and strongly immunoreactive cell morphologies, occurred earlier for astrocytes (i.e. 6–24 h post-injury) as compared to microglia (i.e. 72 h post-injury). A similar but delayed gliotic response was also observed in the thalamus. In fact, a strong astrocyte response (i.e. GFAP reactivity) in the thalamus at 72 h corresponded to extensive fiber degeneration observed at day 7 post-injury indicating that GFAP may be a useful marker for predicting an impending degeneration of tissue fibers in the brain.

## Competing interests

The author(s) declare that they have no competing interests.

## Authors' contributions

AJW designed the study, analyzed data, prepared the figures and wrote the manuscript. HHW performed the QRT-PCR experiments, analyzed data, and contributed to the preparation of the manuscript. JRD and FCT both participated in study design and preparation of the manuscript. All authors have read and approved the final manuscript.
